# Clinical effectiveness of efgartigimod in a broad population of patients with generalized myasthenia gravis: subgroup analyses from a randomized, double‑blind, placebo‑controlled, phase 3 trial (ADAPT)

**DOI:** 10.1007/s00415-026-13877-z

**Published:** 2026-06-03

**Authors:** James F. Howard, Francesco Saccà, Sarah Hoffmann, Shahram Attarian, Jan L. De Bleecker, Jon Beauchamp, Edward Brauer, René Kerstens, John Vissing, Andreas Meisel, The ADAPT Study Group

**Affiliations:** 1 Department of Neurology, University of North Carolina at Chapel Hill, Chapel Hill, NC USA; 2 GENESIS Department, Federico II University, Naples, Italy; 3 Department of Neurology With Experimental Neurology, Integrated Myasthenia Gravis Center, Neuroscience Clinical Research Center, Charité Universitätsmedizin Berlin, Berlin, Germany; 4 Reference Center for Neuromuscular Diseases and ALS, Timone University Hospital, Marseille, Aix-Marseille, France; 5 Faculty of Medicine, Aix-Marseille, Marseille, France; 6 Department of Neurology, University Hospital Ghent, Ghent, Belgium; 7 argenx, Ghent, Belgium; 8 Copenhagen Neuromuscular Center, Rigshospitalet, University of Copenhagen, Copenhagen, Denmark

**Keywords:** Efficacy, Efgartigimod, Generalized myasthenia gravis, Safety, Subgroups

## Abstract

**Background:**

The ADAPT phase 3 trial (NCT03669588) showed efgartigimod was well tolerated and efficacious in acetylcholine receptor antibody–positive (AChR-Ab +) patients with generalized myasthenia gravis (gMG). This analysis utilized data from the ADAPT trial to investigate the efficacy and safety of efgartigimod in different patient subgroups.

**Methods:**

ADAPT included a broad population of AChR-Ab + participants who received a stable dose of ≥ 1 (any) treatment for gMG and were randomized 1:1 to efgartigimod (10 mg/kg) or placebo (administered as four once-weekly infusions per cycle) for 26 weeks. Myasthenia Gravis Activities of Daily Living (MG-ADL) and Quantitative Myasthenia Gravis (QMG) responder rates in cycles 1 and 2, and rates of treatment-emergent adverse events (TEAEs) were analyzed in the following patient subgroups: history of nonsteroidal immunosuppressive treatment (NSIST), concomitant use of gMG treatments throughout the study, and baseline patient and disease characteristics, including time since diagnosis, age, baseline MG-ADL score, body mass index (BMI), sex, and prior thymectomy.

**Results:**

In total, 129 AChR-Ab + participants were included (efgartigimod, *n* = 65; placebo, *n* = 64). Across all subgroups, higher MG-ADL and QMG responder rates were observed in participants treated with efgartigimod versus placebo during cycles 1 and 2. Rates of TEAEs were similar between participants treated with efgartigimod and placebo, regardless of concomitant gMG treatment type.

**Conclusion:**

Similar to outcomes observed in the ADAPT overall population, efgartigimod was well tolerated and efficacious across a broad range of patients, regardless of NSIST treatment history, concomitant use of gMG treatments, or baseline patient and disease characteristics.

**Trial registration:**

ClinicalTrials.gov: NCT03669588 (registered September 13, 2018).

**Supplementary Information:**

The online version contains supplementary material available at 10.1007/s00415-026-13877-z.

## Introduction

Generalized myasthenia gravis (gMG) is a rare, chronic, neuromuscular, autoimmune disease, mediated by pathogenic immunoglobulin (Ig)G autoantibodies [1–3]. It causes debilitating and potentially life-threatening skeletal muscle weakness, including combinations of ocular (ptosis, diplopia) and generalized symptoms such as difficulty in speaking or swallowing, shortness of breath, and/or weakness in muscles of the limbs or neck [2, 3]. Despite current treatments, many patients with gMG continue to experience debilitating symptoms that negatively impact their quality of life (QoL) [2, 4–6]. Up to 20% of patients with gMG may experience myasthenic crisis, a life-threatening respiratory failure event requiring mechanical ventilation [3, 7].

gMG is a heterogeneous disease affected by various factors, including ethnicity, sex, age at disease onset, and time since diagnosis; therapeutic strategies often differ across geographical regions [8]. The heterogeneous nature of the condition may lead to uncertainty regarding expected treatment response in patients with different baseline characteristics who are receiving various concomitant therapies. Commonly used treatments, including corticosteroids and nonsteroidal immunosuppressive treatments (NSISTs), may fail to provide sufficient symptom relief and are associated with burdensome side effects [9, 10]. Many NSISTs have a delayed onset of action (up to 12 months after initiation) and may take months to demonstrate a significant beneficial effect in some patients [11]. About 50% of patients experience side effects with current standard treatments and have to stop medication due to side effects [12]. Commonly used treatments may therefore be insufficient to achieve current treatment goals and to ensure sufficient preservation or restoration of patient QoL [10, 13]. There is a clear unmet need for fast-acting, effective, and well-tolerated treatments for patients with gMG [1, 2, 14].

The majority (~ 85%) of patients with gMG have IgG autoantibodies against the skeletal acetylcholine receptor (AChR), and ~ 5% and ~ 1–5% of patients are seropositive for autoantibodies against muscle-specific tyrosine kinase and low-density lipoprotein receptor–related protein 4, respectively [1]. Approximately 10% of patients have no detectable antibodies (Abs) using current assay methods (i.e. are seronegative) [2, 15]. The pathogenic mechanisms of action of AChR autoantibodies include functional blockade of the AChR and accelerated internalization, leading to reduced AChR density and activation of the complement system, events that lead to the failure of neuromuscular junction transmission [15–19]. The neonatal Fc receptor (FcRn) is an MHC class I–like molecule that recycles IgG, extending IgG half-life ~ 4 times relative to other Igs that are not recycled by FcRn (i.e. IgA, IgD, IgE, and IgM) [20–22]. By maintaining high levels of circulating IgG Abs, FcRn prolongs the availability of pathogenic IgG autoantibodies in patients with gMG [21, 22].

Efgartigimod is a human IgG1 Ab Fc fragment that has been engineered for increased affinity to FcRn compared with endogenous IgG and is uniquely composed of the only part of the IgG Ab that normally binds FcRn [21]. Efgartigimod retains the characteristic pH-dependent binding of IgG Abs, allowing efgartigimod to be released from FcRn, extending pharmacological action in circulation [21, 22]. Efgartigimod selectively reduces IgG by blocking FcRn-mediated IgG recycling [21, 22]. FcRn blockade by efgartigimod thereby causes a reduction in circulating IgG Abs following lysosomal degradation via the lysosome [21, 22]. Efgartigimod significantly reduced concentrations of all IgG subtypes without impacting IgG production, decreasing levels of other Igs (i.e. IgA, IgD, IgE, and IgM), increasing low-density lipoprotein cholesterol, or decreasing albumin, which is also recycled by FcRn [21, 23–25].

ADAPT (NCT03669588) was a randomized, double-blind, placebo-controlled, international, multicenter, pivotal phase 3 trial that investigated the effects of intravenous efgartigimod in adult participants with either AChR Ab–positive (AChR-Ab +) or AChR Ab–negative (AChR-Ab −) gMG [22]. ADAPT met its primary endpoint, demonstrating greater MG-ADL responder rates in AChR-Ab + participants with gMG who were treated with efgartigimod vs. those treated with placebo (68% vs. 30%, respectively; *p* < 0.0001) in cycle 1 [22]. Efgartigimod was well tolerated in participants with gMG, with 152 (91%) of 167 participants completing treatment [22]. In this report, we present subgroup analyses of the ADAPT trial, exploring the efficacy and safety of efgartigimod in AChR-Ab + participants analyzed according to prior NSIST exposure, concomitant use of gMG treatments throughout the study, and baseline patient and disease characteristics, including age, sex, time since diagnosis, and baseline MG-ADL score. This type of analysis is clinically important as subgroup-level evidence is relevant for clinical decision-making, especially when moving toward earlier or individualized use of specific therapies in gMG.

## Methods

### Study design and participants

The primary analyses from ADAPT have previously been published [22]. The study protocol and all amendments received approval by the institutional review board or ethics committee at each participating center (for full list, see Supplementary Table 1). The trial was conducted according to the principles outlined in the Declaration of Helsinki. Participants were eligible for ADAPT if they were aged ≥ 18 years with gMG, with or without AChR Abs, categorized as Myasthenia Gravis Foundation of America (MGFA) class II–IV, and they had a MG-ADL score of ≥ 5 (with > 50% of the MG-ADL score due to nonocular symptoms). Inclusion criteria required participants to be receiving a stable dose of at least one treatment for gMG (i.e. NSISTs, steroids, or acetylcholinesterase [AChE] inhibitors, either alone or in combination). There was no requirement to have received and/or failed any specific gMG treatment (i.e. not a refractory gMG population), nor were participants excluded based on time since diagnosis or history of prior thymectomy (if prior thymectomy ≥ 3 months prior to screening). The primary endpoint was the proportion of AChR-Ab + participants who were MG-ADL responders in the first treatment cycle. A key secondary endpoint was the proportion of AChR-Ab + participants who were Quantitative Myasthenia Gravis (QMG) responders [22].

MG-ADL responders were defined as participants who had at least a 2-point improvement (reduction) in total MG-ADL score that was sustained for ≥ 4 consecutive weeks, with the first improvement occurring by week 4 of the treatment cycle (1 week after the fourth infusion). QMG responders were defined as having a ≥ 3-point improvement (reduction) in total QMG score for ≥ 4 consecutive weeks, with the first improvement occurring by week 4 of the cycle (1 week after the fourth infusion).

Participants were randomly assigned 1:1 to receive either efgartigimod (10 mg/kg) or placebo during a 26-week treatment period. Participants received four once-weekly infusions of study drug per cycle. Following an initial cycle, subsequent cycles were administered according to individual clinical evaluation when the following criteria were met: ≥ 8 weeks since initiation of previous cycle; MG-ADL score of ≥ 5; and the patient was an MG-ADL responder who no longer had a clinically meaningful decrease (MG-ADL total score ≥ 2-point improvement) compared with baseline [22]. Due to this adaptive dosing design, the number of participants receiving 3 treatment cycles during the 26-week study period was limited; therefore, subgroup analyses were focused on cycles 1 and 2, which included the majority of evaluable participants and captured the primary and key secondary endpoints.

### Statistical analyses

Like the primary analyses, all subgroup analyses were performed on the AChR-Ab + patients from the modified intent-to-treat population. Differences in efficacy were evaluated for several subgroups during the first two treatment cycles based on two efficacy endpoints: the proportion of MG-ADL responders (patient-reported, physician-recorded outcome measure) [26] and the proportion of QMG responders (physician-assessed, including quantitative measures).

The evaluation of differences in efficacy was performed for the following subgroups: history of (prior) treatment with an NSIST (any or no); concomitant use of gMG treatment during the study (any gMG treatment, any or no steroids, any or no NSISTs, only AChE inhibitors); and patient and disease characteristics, including time since diagnosis (0 to < 3 or ≥ 3 years), baseline MG-ADL score (5–8 or ≥ 9), sex, age (< 65 or ≥ 65 years old), body mass index (BMI; < 30 or ≥ 30 kg/m^2^), and prior thymectomy (yes or no). For each subgroup, the proportions of responders per treatment arm were tabulated together with the difference between the proportions (efgartigimod minus placebo) with the asymptotic standard errors and 95% Wald confidence limits. Forest plots were created to facilitate interpretation, and corresponding statistical results are presented in the figures.

Safety was assessed by the incidence of treatment-emergent adverse events (TEAEs), including a specific assessment of TEAEs of special interest corresponding to the Medical Dictionary for Regulatory Activities System Organ Class of “Infections and Infestations.” These safety results were summarized for subgroups based on concomitant use of gMG treatments during the study (any gMG treatment, any or no steroids, any or no NSISTs, only AChE inhibitors).

## Results

### Participants

In total, 167 participants with gMG were randomized and treated during the ADAPT trial, of whom 129 (77.2%) were AChR-Ab + (efgartigimod, *n* = 65; placebo, *n* = 64).

Baseline patient and disease characteristics are presented in Table 1. These were well balanced and comparable between the efgartigimod and placebo groups, with the exception of the proportion of participants who had undergone thymectomy, which was higher in participants treated with efgartigimod vs. placebo (69.2% vs. 46.9%). The proportion of participants receiving only AChE inhibitors at baseline was also higher in the efgartigimod group compared with the placebo group (20.0% vs. 9.4%). At screening, the majority of participants presented as MGFA class III, were younger than 65 years old, and had a gMG disease duration (time since diagnosis) of ≥ 3 years, with the mean (range) time since diagnosis being 9.7 (1.0–45.3) years for efgartigimod-treated participants and 8.9 (0.2–36.1) years for placebo-treated participants. Median (interquartile range) baseline MG-ADL and QMG scores were 9.0 (2.0) and 16.0 (6.0), respectively, for the efgartigimod-treated participants, and 8.0 (3.0) and 15.5 (6.0), respectively, for placebo-treated participants. In the efgartigimod-treated participants, 61.5% had an MG-ADL score ≥ 9 at baseline vs. 46.9% of participants in the placebo group.

**Table 1:** Baseline patient and disease characteristics of AChR-Ab + participants

	Efgartigimod (*n* = 65)	Placebo (*n* = 64)
Age, mean years (SD)	44.7 (15.0)	49.2 (15.5)
< 65, *n* (%)	57 (87.7)	51 (79.7)
≥ 65, *n* (%)	8 (12.3)	13 (20.3)
Female, *n* (%)	46 (70.8)	40 (62.5)
Time since diagnosis, mean years (range)	9.7 (1.0–45.3)	8.9 (0.2–36.1)
0–3 years, *n* (%)	14 (21.5)	17 (26.6)
≥ 3 years, *n* (%)	51 (78.5)	47 (73.4)
MGFA Class at screening, *n* (%)		
II	28 (43.1)	25 (39.1)
III	35 (53.8)	36 (56.3)
IV	2 (3.1)	3 (4.7)
MG-ADL score		
Mean (range)	9.0 (5.0–15.0)	8.6 (5.0–16.0)
Median (IQR)	9.0 (2.0)	8.0 (3.0)
MG-ADL score at baseline, *n* (%)		
5–8	25 (38.5)	34 (53.1)
≥ 9	40 (61.5)	30 (46.9)
9–12	34 (52.3)	28 (43.8)
13–16	6 (9.2)	2 (3.1)
QMG score		
Mean (range)	16.0 (4.0–28.0)	15.2 (6.0–24.0)
Median (IQR)	16.0 (6.0)	15.5 (6.0)
Thymectomy, *n* (%)	45 (69.2)	30 (46.9)
Any prior use of NSIST, *n* (%)	47 (72.3)	43 (67.2)
Azathioprine	29 (44.6)	29 (45.3)
Ciclosporin	15 (23.1)	12 (18.8)
Mycophenolate mofetil	11 (16.9)	6 (9.4)
Mycophenolate sodium	1 (1.5)	0
Cyclophosphamide	3 (4.6)	5 (7.8)
Tacrolimus	5 (7.7)	3 (4.7)
Methotrexate	0	4 (6.3)
No prior use of NSIST	18 (27.7)	21 (32.8)
Concomitant use of gMG treatments at study baseline, *n* (%)	65 (100.0)	64 (100.0)
AChE inhibitor only	13 (20.0)	6 (9.4)
Any AChE inhibitor	57 (87.7)	57 (89.1)
Any steroid	46 (70.8)	51 (79.7)
Any NSIST	40 (61.5)	37 (57.8)
Steroid and NSIST	34 (52.3)	31 (48.4)

*AChE* acetylcholinesterase, *AChR-Ab* + acetylcholine receptor antibody–positive, *gMG* generalized myasthenia gravis, *IQR* interquartile range, *MG-ADL* Myasthenia Gravis Activities of Daily Living, *MGFA* Myasthenia Gravis Foundation of America, *NSIST* nonsteroidal immunosuppressive treatment, *QMG* Quantitative Myasthenia Gravis, *SD* standard deviation

Most participants received some form of NSIST throughout the study, with around half of the participants receiving both a steroid and an NSIST. Eighteen (27.7%) efgartigimod-treated participants and 21 (32.8%) placebo-treated participants had no history of prior treatment with an NSIST.

### MG-ADL and QMG responder rates by use of gMG treatments

**Fig. 1:**
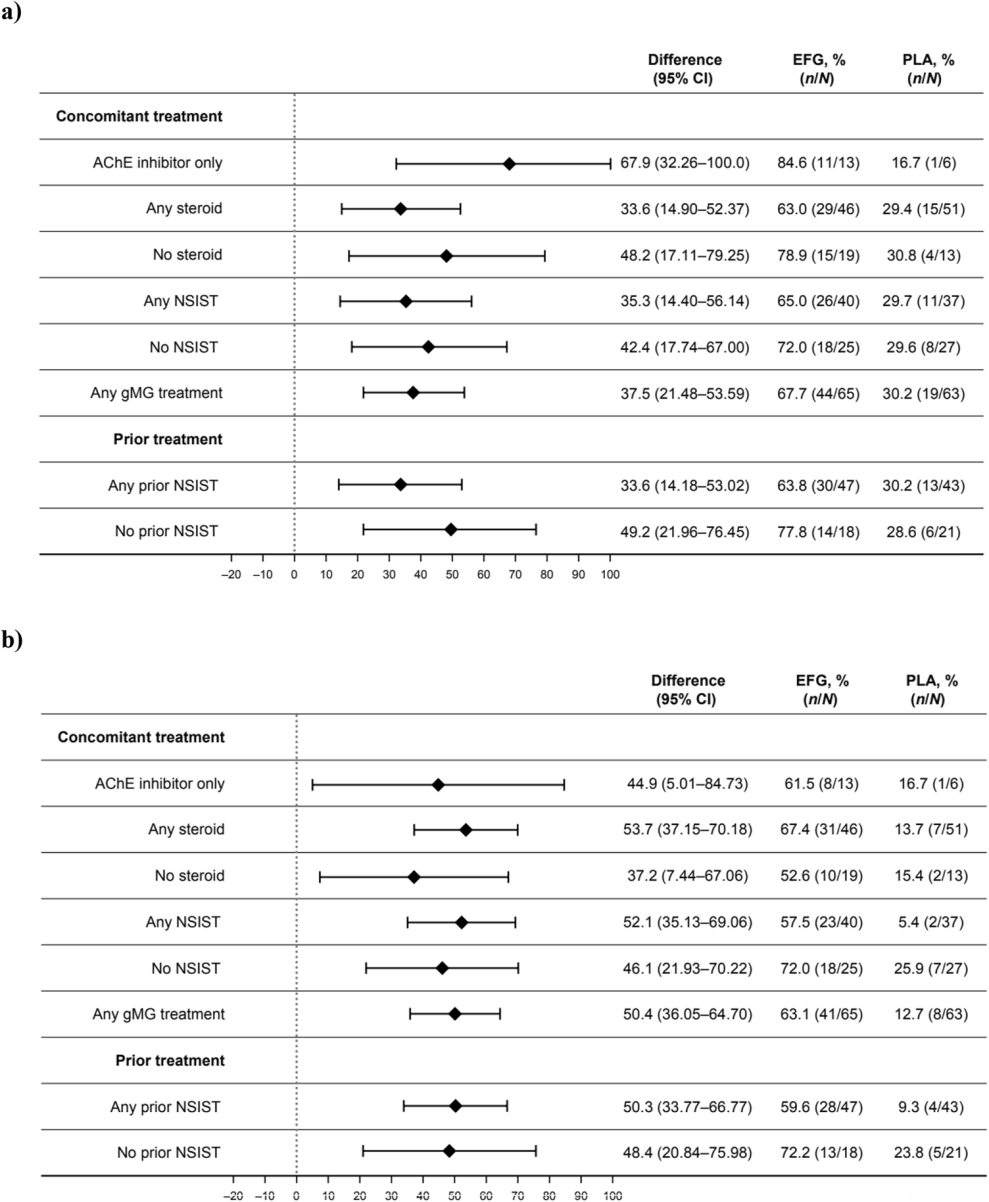
Proportion of **a** MG-ADL and **b** QMG responders in cycle 1 by use of gMG treatments during the original study or NSISTs prior to study initiation. One patient in the placebo group was not receiving any concomitant gMG treatment during the original study and was excluded from respective analyses. *AChE* acetylcholinesterase, *CI* confidence interval, *EFG* efgartigimod, *gMG* generalized myasthenia gravis, *MG-ADL* myasthenia gravis activities of daily living, *NSIST* nonsteroidal immunosuppressive treatment, *PLA* placebo, *QMG* quantitative myasthenia gravis

In all subgroups analyzed according to gMG treatment received concomitant to the study drug, including participants who did or did not receive any steroid or NSIST or who received AChE inhibitors only, significantly higher MG-ADL and QMG responder rates were observed in participants treated with efgartigimod vs. placebo during cycle 1 (Fig. 1). Regardless of history of prior NSIST treatment, significantly higher MG-ADL and QMG responder rates were observed in participants treated with efgartigimod vs. placebo during cycle 1 (Fig. 1). Consistent with cycle 1, MG-ADL and QMG responder rates in cycle 2 were greater, and generally significantly greater, in participants treated with efgartigimod vs. those treated with placebo, regardless of concomitantly received gMG treatments or history of prior NSIST treatment (Supplementary Fig. 1). MG-ADL and QMG responder rates by subgroups based on use of gMG treatments were also greater, and typically significantly greater, in those treated with efgartigimod vs. placebo when analyzing participants who responded in both cycle 1 and cycle 2 (Supplementary Fig. 2).

### MG-ADL and QMG responder rates by baseline patient and disease characteristics

**Fig. 2:**
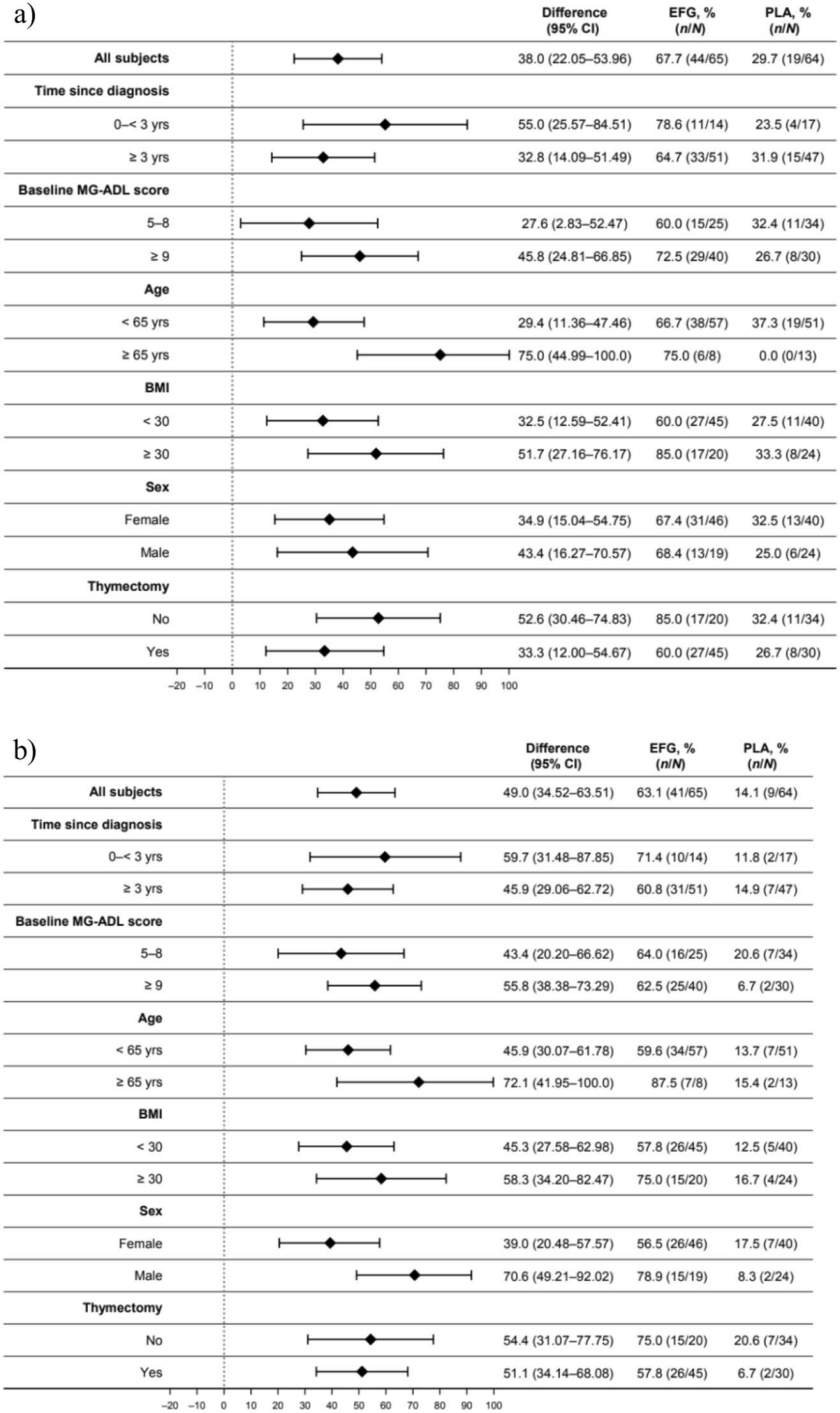
Proportion of **a** MG-ADL and **b** QMG responders in cycle 1 by baseline patient and disease characteristics. *BMI* body mass index, *CI* confidence interval, *EFG* efgartigimod, *MG-ADL* myasthenia gravis activities of daily living, *PLA* placebo, *QMG* quantitative myasthenia gravis

Regardless of age, sex, and BMI at baseline, MG-ADL and QMG responder rates were significantly higher in participants treated with efgartigimod in cycle 1 vs. those treated with placebo (Fig. 2). Similarly, significantly higher proportions of MG-ADL and QMG responder rates were reported in the efgartigimod group in cycle 1 vs. the placebo group, regardless of time since diagnosis, baseline MG-ADL score, or whether the patient had undergone thymectomy (Fig. 2).

Consistent with cycle 1, MG-ADL and QMG responder rates in cycle 2 were greater, and typically significantly greater, in participants treated with efgartigimod vs. those treated with placebo, regardless of all baseline patient and disease characteristics (Supplementary Fig. 3). MG-ADL and QMG responder rates in all subgroups defined by baseline patient and disease characteristics were also greater, and generally significantly greater, in those treated with efgartigimod vs. placebo when analyzing participants who responded in both cycle 1 and cycle 2 (Supplementary Fig. 4).

### Safety according to concomitant gMG treatments

The rates of TEAEs, serious TEAEs, or severe TEAEs were similar in participants treated with efgartigimod vs. placebo when analyzed according to any gMG treatment received concomitant to the study drug, with no notable differences when analyzing by use of steroids, NSISTs, or AChE inhibitors (Supplementary Table 2). The proportion of participants who experienced at least one TEAE of special interest (infection) was generally greater in the efgartigimod group (44.6%) vs. the placebo group (33.3%) when analyzed according to any concomitantly received gMG treatment (Supplementary Table 2). No notable differences in rates of TEAEs of special interest were observed in participants treated with efgartigimod vs. placebo when analyzing according to concomitant use of steroids, NSISTs, or AChE inhibitors (41.3–47.5% vs. 29.7–31.4%, respectively; Supplementary Table 2). The vast majority of infections were mild to moderate in severity, with severe infections reported in two (2.4%) efgartigimod-treated participants (influenza and pharyngitis) and one (1.2%) placebo-treated participant (upper respiratory tract infection). TEAEs that led to withdrawal of the study drug were similar between participants treated with efgartigimod vs. placebo when analyzed according to any concomitantly received gMG treatment (3.1% vs. 4.8%, respectively; Supplementary Table 2), with no notable differences when analyzing by concomitant use of any steroid, NSIST, or AChE inhibitor. There were no fatalities reported.

## Discussion

As the disease landscape evolves for gMG, individual patient characteristics should be considered in order to tailor treatment approaches and provide the best clinical benefit as early as possible in the disease course of each patient. Emerging treatment options for patients with gMG may help broaden the therapeutic landscape beyond the use of traditional, non-specific immunosuppressants and toward more targeted therapies that are effective across a broad range of patients regardless of their history of disease or treatments [9].

The ADAPT study demonstrated that selective IgG reduction via efgartigimod was both efficacious and well tolerated in participants with gMG [22]. Among the AChR-Ab + participants in ADAPT, a greater proportion of MG-ADL responders was observed following efgartigimod treatment vs. placebo in cycle 1 (68% vs. 30%, respectively; p < 0.0001) [22]. The interim analysis of the open-label extension study, ADAPT + (NCT03770403), further demonstrated that treatment with efgartigimod was well tolerated and that consistent and repeatable clinical improvements in participants with gMG were achieved across multiple treatment cycles [27].

The analysis presented here showed that similar MG-ADL and QMG responder rates in cycle 1 were observed across all subgroups analyzed, suggesting the efficacy of efgartigimod is independent of baseline patient and disease characteristics and history of gMG treatments received. This clinical efficacy was observed in patient subgroups associated with early gMG disease (such as those with a disease duration [time since diagnosis] of < 3 years, those with no history of thymectomy, or those with history of treatment with AChE inhibitors only), as well as subgroups associated with long-term or severe gMG disease (such as those with a time since diagnosis of ≥ 3 years or a baseline MG-ADL score of ≥ 9, respectively). In support of these findings, a previous subgroup analysis found that treatment with the complement C5 inhibitor ravulizumab led to improved clinical outcomes regardless of the time from diagnosis [28]. Differences in MG-ADL responder rates observed in efgartigimod- and placebo-treated participants with a study baseline MG-ADL score of 5‒8 were slightly lower vs. those observed in participants with a study baseline MG-ADL score of ≥ 9 in both cycles 1 and 2. This may be attributed to a greater difficulty in meeting the criteria for a 2-point improvement in MG-ADL score in those with relatively low baseline scores vs. those with an MG-ADL score of ≥ 9. Overall, these data suggest efgartigimod may be a suitable treatment option for patients in both the early and late stages of their gMG disease course.

In the ADAPT and ADAPT + trials, efgartigimod demonstrated repeatability of effect across multiple treatment cycles [22, 27]. This clinical effectiveness across treatment cycles extends to the subgroups analyzed here, where greater MG-ADL responder rates were also observed during cycle 2 in participants treated with efgartigimod vs. placebo. Similarly, QMG responder rates in cycle 2 were greater across all subgroups analyzed when compared with placebo, albeit numerically lower than responder rates observed in cycle 1. This may be attributed to the use of a patient’s MG-ADL score to define the initiation of a second cycle of treatment. At the time of starting a second treatment cycle, 35.3% of participants who received efgartigimod in cycle 1 continued to have a ≥ 3-point improvement from study baseline in QMG score and could not be expected to achieve an additional 3-point improvement to meet the QMG responder criteria in cycle 2. In these analyses, placebo MG-ADL and QMG responder rates were also consistently, and typically significantly, lower than for efgartigimod-treated participants in cycle 1 and cycle 2. This sustained treatment effect aligns with findings from a separate post hoc analysis of participants in ADAPT which demonstrated that the overall time in response (according to MG-ADL and QMG scores) was significantly greater in efgartigimod-treated participants vs. those treated with placebo [29]. Taken together, these data show that a greater proportion of efgartigimod-treated participants than placebo-treated participants experienced sustained MG-ADL and QMG responses across successive treatment cycles, demonstrating the long-term clinical benefit of efgartigimod.

As more immunotherapeutics emerge for patients with gMG, monitoring of disease activity, severity, and response to treatment is recommended to assess whether therapeutic goals are met by the current treatment of choice [13]. Modern therapeutic goals in gMG aim for the best possible disease control and restoration of patient QoL, which can be assessed using MG-ADL and QMG scores, although the Myasthenia Gravis Quality of Life 15-Item Questionnaire is a more recognized measure [30]. Establishing whether therapeutic goals are achieved may be hindered by the potential placebo effect that has been noted in individuals with gMG. A meta-analysis of randomized and placebo-controlled trials reported a small effect of placebo on MG-ADL score (range of − 0.9 to − 2.3 across six assessments) [31]. Similarly, an additional meta-analysis reported small but significantly lower responses with placebo vs. active treatment based on QMG scores [32]. This potential effect may be due to the use of concomitant treatment in clinical trials, but data from trials investigating C5 and FcRn inhibitors have also consistently shown a strong placebo effect in the first 1 to 4 weeks after the start of treatment [23, 33]. Thus, the impact of concomitant treatment may be minor, but subsequent studies are required.

A previous study found that females have a poorer response to standard gMG treatments than males, with less improvement in both objective and patient-reported outcomes [34]. In the current analyses, females showed greater MG-ADL and QMG responder rates following treatment with efgartigimod vs. those who received placebo (a difference of 34.9% [95%CI 15.04–54.75%] and 39.0% [95%CI 20.48–57.57%], respectively), despite both MG-ADL and QMG responder rates being numerically lower than those observed in males. MG-ADL values in males treated with efgartigimod were 68.4%, compared to 67.4% for females, and 25.0% for males treated with placebo, compared with 32.5% for females. QMG values for males treated with efgartigimod were 78.9%, compared with 56.6% for females, and 8.3% for males treated with placebo, compared with 17.5% for females. Observing both the patient-reported MG-ADL score and the physician-assessed QMG score, these results suggest efgartigimod is efficacious in both females and males.

The ADAPT and ADAPT + trials showed efgartigimod was well tolerated, with most TEAEs being mild or moderate in severity [22, 27]. In these analyses, the rates of TEAEs observed in participants treated with efgartigimod were similar to those observed in participants treated with placebo. Rates of TEAEs observed in efgartigimod- and placebo-treated participants were also consistent between subgroups analyzed according to concomitant use of any gMG treatment, with no notable differences following the use of steroids, NSISTs, or AChE inhibitors. It should be noted that infections are not expected to be more frequent in subjects treated with steroids or NSISTs, regardless of the treatment arm, and the subgroups’ sample sizes are likely insufficient to reliably detect differences in rare TEAEs. Rates of TEAEs that led to withdrawal from the study drug were also similar between participants treated with efgartigimod and placebo when analyzed according to concomitant use of any gMG treatment. These observations suggest efgartigimod is well tolerated regardless of concomitant use of other gMG treatment, with no additional safety concerns or limitations arising from the use of steroids, NSISTs, or AChE inhibitors while receiving efgartigimod.

In these analyses, the rates of infection (mostly mild to moderate in severity) observed according to any concomitantly received gMG treatment were similar to those observed in the overall population from ADAPT (46% efgartigimod, 37% placebo). No notable increase in infection rates was observed in efgartigimod-treated participants who received concomitant steroids, NSISTs, or AChE inhibitors. The mechanism of action of efgartigimod facilitates selective IgG reduction that substantially, but incompletely, reduces IgG levels (mean maximal reductions of ~ 60%) without impacting IgG production or other parts of the immune system, such as albumin [22, 23]. This selective IgG reduction allows efgartigimod-treated patients to mount an immune response, as evidenced in ADAPT + where antigen-specific IgG responses to vaccinations were observed [35].

In addition to these data supporting efgartigimod’s effectiveness and tolerability across patient subgroups, a secondary analysis of the ADAPT study showed significant improvements in health-related QoL that were rapid and substantial, representing a broader benefit of efgartigimod treatment beyond the relief of gMG symptoms [22, 36].

Strengths of the ADAPT trial included the enrollment of a broad and representative population of patients with gMG, which permitted a wide variety of subgroup analyses to be performed, and use of validated instruments enabling assessment of both symptom burden and disease severity. Limitations of these analyses include the relatively low patient number for some of the defined subgroups, reflecting the post hoc nature of these analyses, and the fact that these subgroup analyses were not predefined, precluding evaluation of statistical significance. No adjustment for multiplicity was applied, and therefore the results of the subgroup analyses should be interpreted as exploratory. In addition, as with all randomized controlled trials, the study population was selected according to predefined eligibility criteria, which may limit the generalizability of these findings to routine clinical practice. As such, real-world studies are needed to further evaluate the effectiveness of efgartigimod across patient subgroups in broader clinical settings. While ADAPT had a relatively short study duration of 26 weeks, the published interim results from the ADAPT + open-label extension study further corroborate the clinical effectiveness and long-term safety and tolerability of efgartigimod [25, 27].

## Conclusion

We have demonstrated the efficacy and safety of efgartigimod in a variety of clinically relevant subgroups, including those early and later in their disease course, which indicates that efgartigimod could be considered as a potential therapy for gMG regardless of the treatments received and baseline patient and disease characteristics. These results build on the findings from the overall population described in the pivotal publication. Efgartigimod offers improvements to QoL through relief of disease and treatment burdens, presenting an opportunity to shift the treatment paradigm toward the use of specific therapies earlier in the disease course and across a broad range of patients.

## Supplementary Information

Below is the link to the electronic supplementary material.

Supplementary file1 (DOCX 706 KB)

## Data Availability

argenx is committed to responsible data sharing regarding the clinical trials it funds. Included in this commitment is access to anonymized individual-level and trial-level data (analysis data sets), and other information (e.g., protocols and clinical study reports), as long as the trial is not part of an ongoing or planned regulatory submission. These clinical trial data can be requested by qualified researchers who engage in rigorous independent scientific research, and they will only be provided after review and approval of a research proposal and statistical analysis plan and the execution of a data sharing agreement. Data requests can be submitted at any time, and the data will be accessible for 12 months. Requests can be submitted to esr@argenx.com.
