# Supplementary material for: Clinical effectiveness of efgartigimod in a broad population of patients with generalized myasthenia gravis: subgroup analyses from a randomized, double‑blind, placebo‑controlled, phase 3 trial (ADAPT)
Source: J Neurol. 2026 Jun 3;273(6):363. doi: 10.1007/s00415-026-13877-z (PMC13233976; doi:10.1007/s00415-026-13877-z)
Supplement: Supplementary file 1 — Supplementary file1 (DOCX 706 KB) [file 415_2026_13877_MOESM1_ESM.docx]

**Supplementary Material**

Clinical effectiveness of efgartigimod in a broad population of patients with generalised myasthenia gravis: subgroup analyses from a randomised, double‑blind, placebo‑controlled, phase 3 trial (ADAPT)

James F. Howard Jr^1^, Francesco Saccà^2^, Sarah Hoffmann^3^, Shahram Attarian^4^, Jan L. De Bleecker^5^,
Jon Beauchamp^6^, Edward Brauer^6,^*, René Kerstens^6^, John Vissing^7^, Andreas Meisel^3^, and the ADAPT Study Group

^1^ Department of Neurology, University of North Carolina at Chapel Hill, Chapel Hill, NC, USA

^2^ GENESIS Department, Federico II University, Naples, Italy

^3^ Department of Neurology with Experimental Neurology, Integrated Myasthenia Gravis Center, Neuroscience Clinical Research Center, Charité Universitätsmedizin Berlin, Berlin, Germany

^4^ Reference Center for Neuromuscular Diseases and ALS, Timone University Hospital, Marseille; Faculty of Medicine, Aix-Marseille, France

^5^ Department of Neurology, University Hospital Ghent, Ghent, Belgium

^6^ argenx, Ghent, Belgium

^7^ Copenhagen Neuromuscular Center, Rigshospitalet, University of Copenhagen, Copenhagen, Denmark

Correspondence: Andreas Meisel, [andreas.meisel@charite.de](mailto:andreas.meisel@charite.de)

*Affiliation at the time the study was conducted.

**Supplementary Table 1** List of independent ethics committees/institutional review boards

| Investigator Name | Name/Address of Ethics Committee | Name of Chairman |
| --- | --- | --- |
| Prof. dr. Jan De Bleecker | Ethisch Commité UZA  Wilrijkstraat 10  2650 Edegem, Belgium | Prof. Peter  Michielsen |
| Prof. dr. Rudolf Mercelis | Ethisch Commité UZA  Wilrijkstraat 10  2650 Edegem, Belgium | Prof. Peter  Michielsen |
| Dr. Vera Bril | University Health Network  Research Ethics Board (UHN REB), 700 University Avenue, 10/F room, 10-56, Toronto, ON M5G 1Z5, Canada | Dr. Morris  Sherman  (Biomedical) |
| Dr. Angela Genge | McGill University Health Center  Research Ethics Board -  Neuroscience and Psychiatry Panel, 3801 University Street  Montreal, QC H3A 2B4, Canada | Dr. Marie-Josée  Brouillette & Dr.  Judith Marcoux |
| Zaeem Siddiqi, MD, PhD | REB4: Health Research Ethics  Board - Biomedical Panel  308 Campus Tower  8625-112 Street, Edmonton, Alberta T6G 1K8, Canada | Dr. Shane  Kimber |
| Jana Junkerova, MD | Multicentricka eticka komise  Fakultni nemocnice Brno  Jihlavska 20, 625 00 Brno, Czech Republic | PharmDr. Šárka  Kozáková, MBA |
| Michael Tyblova, MD, PhD | Multicentricka eticka komise  Fakultni nemocnice Brno  Jihlavska 20, 625 00 Brno, Czech Republic | PharmDr. Šárka  Kozáková, MBA |
| Stanislav Vohanka, MD, PhD, MBA | Multicentricka eticka komise  Fakultni nemocnice Brno  Jihlavska 20, 625 00 Brno, Czech Republic | PharmDr. Šárka  Kozáková, MBA |
| Prof. Dr. Andreas Meisel | Landesamt für Gesundheit und  Soziales (LAGeSo)  Geschäftsstelle der Ethik-  Kommission des Landes Berlin  Turnstraße 21, 10559 Berlin, Germany | Prof. Dr.  Edeltraut Garbe |
| Dr. John Vissing | Videnskabsetisk Komité – Region, Hovedstaden  Kongens Vaenge 2  3400 Hillerod, Denmark | Mette S. Kjær |
| Dr. Henning Andersen | Videnskabsetisk Komité – Region, Hovedstaden  Kongens Vaenge 2, 3400 Hillerod, Denmark | Mette S. Kjær |
| Dr. Guilhem Sole | Comité de Protection des Personnes, Sud Méditerranée IV  Hôpital St. Eloi, 34295 Montpellier Cedex 5, France | Professeur Jean-  Marc Davy |
| Dr. Shahram Attarian | Comité de Protection des Personnes, Sud Méditerranée IV  Hôpital St. Eloi, 34295 Montpellier Cedex 5, France | Professeur Jean-  Marc Davy |
| Dr. Saiju Jacob | West Midlands - Edgbaston  Research Ethics Committee  The Old Chapel, Royal Standard  Place, NG1 6FS Nottingham, United Kingdom | Mr Paul  Hamilton |
| Dr. Sivakumar Sathasivam | West Midlands - Edgbaston  Research Ethics Committee  The Old Chapel, Royal Standard  Place, NG1 6FS Nottingham, United Kingdom | Mr Paul  Hamilton |
| Temur Margania | Local Ethics Committee of Ltd new Hospitals, 12 Krtsanisi Street, Tbilisi 0114, Georgia | Mikheil  Virsaladze |
| Roman Shakarishvili | Local Ethics Committee of Ltd  Petre Sarajishvili Institute of  Neurology, 13 Tevdore Mgvdeli Street, Tbilisi 0112, Georgia | Dr Maia Jibladze |
| Alexander Tsiskaridze | Local Ethics Committee of Ltd  Pineo Medical Ecosystem  93 Gorgasali Street, Tbilisi 0114, Georgia | Dr Tekle  Kishmaraia |
| László Vécsei, MD | Medical Research Ethics Council  Ethics Committee for Clinical  Pharmacology, Széchenyi István tér 7-8, H-1051 Budapest, Hungary | Prof. Dr.  Zsuszunna Fürst,  MD |
| Csilla Rósza, MD | Medical Research Ethics Council  Ethics Committee for Clinical  Pharmacology, Széchenyi István tér 7-8, H-1051 Budapest, Hungary | Prof. Dr.  Zsuszunna Fürst,  MD |
| Mária Judit Molnár, MD, PhD, DSc | Medical Research Ethics Council  Ethics Committee for Clinical  Pharmacology, Széchenyi István tér 7-8, H-1051 Budapest, Hungary | Prof. Dr.  Zsuszunna Fürst,  MD |
| Dr. Renato Mantegazza | Comitato Etico IRCCS Regione  Lombardia - Sez. Fondaz. IRCCS, Istituto Neurologico Carlo Besta, Via Celoria 11  20133 Milano, Italy | Prof. Alfredo  Gorio |
| Dr. Franceso Saccà | Comitato Etico dell'Università  Federico II, Via Pansini 5  80131 Napoli, Italy | Prof. Claudio  Buccelli |
| Dr. Giovanni Antonini | Comitato Etico Università Sapienza di Roma, Via di Grottarossa, 1035-1039  00189 Roma, Italy | Prof. Giovanni  Spera |
| Dr. Shingo Konno | Toho University Ohashi Medical  Center Institutional Review Board, 2-22-36 Ohashi, Meguro-ku, Tokyo 153-8515, Japan | Professor  Yoshihisa Saida |
| Dr. Akiyuki Uzawa | Chiba University Hospital  Institutional Review Board  1361-Matoba, Kawagoe-shi  Saitama 350-1101, Japan | Professor  Koichiro Tatsumi |
| Dr. Makoto Samukawa | Kindai University Hospital  Institutional Review Board  377-2, Ono-Higashi, Osaka-  Sayama-shi, Osaka 589-8511, Japan | Dr. Tetsuya  Mitsudomi |
| Dr. Kimiaki Utsugisawa | General Hanamaki Hospital  Institutional Review Board  4-28, Kajomachi, Hanamaki-shi  Iwate 025-0075, Japan | Dr. Masashi  Sawada |
| Dr. Yasushi Suzuki | National Hospital Organization  Sendai Medical Center Institutional Review Board  2-11-12 Miyagino,Sendai-shi  Miyagi 983-8520, Japan | Dr. Katsuaki  Ukai |
| Dr. Hiroyuki Murai | International University of Health and Welfare Institutional Review Board  1-24-1, Minami-Aoyama, Minatoku, Tokyo 107-0062, Japan | Professor  Tsutomu  Yamazaki |
| Dr. Masanori Takahashi | Institutional Review Board of Osaka University Hospital  2-15 Yamadaoka, Suita-shi  Osaka 565-0871, Japan | Dr. Haruhiko  Kishima |
| Dr. Tomihiro Imai | Sapporo Medical University  Hospital Institutional Review Board, Minami 1 Nishi 16, Chuo-ku, Sapporo  Hokkaido 060-8543, Japan | Professor Hiroshi  Nakase |
| Dr. Masayuki Masuda | Tokyo Medical University Hospital Institutional Review Board, 6-7-1 Nishishinjuku, Shinjuku-ku  Tokyo 160-0023, Japan | Dr. Haruo Hanyu |
| Dr. Takemori Yamawaki | Hiroshima City Hiroshima Citizens, Hospital Institutional Review Board  7-33 Motomachi, Hiroshima-city  Hiroshima 730-8518, Japan | Masazumi  Okajima |
| Dr. Johannes J.G.M.  Verschuuren | Medisch-Ethische  Toetsingscommissie Leiden Den  Haag Delft (METC LDD)  Albinusdreef 2  2300 RC, Leiden, South Holland  The Netherlands | Prof. Dr. Albert  Dahan |
| Małgorzata Bilińska, MD, PhD | Komisja Bioetyczna przy  Okręgowej Izbie Lekarskiej w  Krakowie, ul. Krupnicza 11a  31-123 Kraków, Poland | Mariusz  Janikowski, M.D. |
| Prof. Andrzej Szczudlik,  MD, PhD | Komisja Bioetyczna przy  Okręgowej Izbie Lekarskiej w  Krakowie, ul. Krupnicza 11a  31-123 Kraków, Poland | Mariusz  Janikowski, M.D. |
| Prof. Anna Kostera-  Pruszczyk, MD, PhD | Komisja Bioetyczna przy  Okręgowej Izbie Lekarskiej w  Krakowie, ul. Krupnicza 11a  31-123 Kraków, Poland | Mariusz  Janikowski, M.D. |
| Lech Szczechowski, MD, PhD | Komisja Bioetyczna przy  Okręgowej Izbie Lekarskiej w  Krakowie, ul. Krupnicza 11a  31-123 Kraków, Poland | Mariusz  Janikowski, M.D. |
| Irina Poverennova, MD, PhD, DMSci., Prof. | Ethics Committee of SBHI "Samara  Regional Clinical Hospital n.a.  V.D.Seredavin"  159, Tashkentskaya str.  443095 Samara, Russia | Dr. Olga  Vladimirovna  Vikulova |
| Prof. Nadezhda Malkova, MD, PhD, DMSci. | Ethics Committee of SBHI of NR, "State Novosibirsk Regional  Clinical Hospital"  130 Nemirovich-Danchenko str.  630087 Novosibirsk, Russia | Dr. Anatoliy  Vasilyevich  Yudanov |
| Prof. Dmitry Pokhabov, MD,  PhD, DMSc., | Ethics Committee of FSBI "Federal  Siberian Scientific and Clinical  Center of Federal Medical and  Biological Agency"  26, Kolomenskaya str.  660037 Krasnoyarsk, Russia | Dr. I.V.  Krasnopeyeva |
| Ass. Prof. Elena Antipenko, DMSc. | Local Ethics Committee of SBHI  NNR "Nizhny Novgorod Region  Clinical Hospital n.a. N.A.  Semashko"  190 Rodionova str.  603126 Nizhny Novgorod, Russia | Borovkov N. N. ,  DMSc., Professor |
| Prof. Sergey Kotov, DMSci. | IEC of SBHI of Moscow Region  "Moscow Regional Scientific  Research Clinical Institute n.a. M.F.  Vladimirsky"  61/2, Shchepkina str.  129110 Moscow, Russia | T.A. Britvin,  DMSc. |
| Dr. Stojan Peric | Ethics Committee of Serbia  Vojvode Stepe 458  11000 Belgrade, Serbia | Prof. Andrija  Bogdanovic,  MD, PhD |
| Said R. Beydoun, MD, FAAN | University of Southern California  Institutional Review Board  1640 Marengo Street, Suite 700  Los Angeles, CA 90033  USA | Darcy V. Spicer,  MD |
| James F. Howard Jr., MD | The University of North Carolina at Chapel Hill Office of Human  Research Ethics  720 Martin Luther King Jr Blvd.  Building #385, Second Floor  Chapel Hill, NC 27599  USA | Dr. David Weber  (Biomedical A) |
| Tahseen Mozaffar, MD | University of California, Irvine -  Office of Research  141 Innovation Drive, Suite 250  Irvine, CA 92697  USA | Kenneth Linden,  M.D., Ph.D. |
| Tuan H. Vu, MD | Western Institutional Review Board (WIRB)  1019 39th Avenue SE, Suite 120  Puyallup, WA 98374  USA | Glenn Veit, JD,  CIP |
| Sarah Jones, MD | Western Institutional Review Board (WIRB)  1019 39th Avenue SE, Suite 120  Puyallup, WA 98374  USA | Glenn Veit, JD,  CIP |
| Tulio E. Bertorini, MD | Copernicus Group IRB  5000 CentreGreen Way, Suite 200  Cary, NC 27513  USA | Glenn Veit, JD,  CIP |
| Ratna K. Bhavaraju-Sanka, MD | University of Texas Health Science  Center at San Antonio Institutional  Review Board  MC 7830, 7703 Floyd Curl Dr.  San Antonio, TX 78229  USA | Kimberly K.  Summers,  PharmD -  Director,  Research  Protection  Programs |
| Katherine Ruzhansky, MD | MUSC Office of Research Integrity  - Institutional Review Board for  Human Research  19 Hagood Avenue, Suite 601  Charleston, SC 29425  USA | David Lewin,  M.D. |
| Laurie Gutmann, MD | The University of Iowa Institutional  Review Board (Human Subject's  Office)  Hardin Library for the Health  Sciences  600 Newton Rd., Suite 105  Iowa City, IA 52242  USA | Andy Bertolatus,  M.D. (primary)  Catherine  Woodman, M.D.  Bill McGinnis,  M.D.  Douglas L.  Somers, M.D.  Dixie Ecklund,  RN, MSN, MBA |
| Chafic Karam, MD | Oregon Health & Science  University Institutional Review  Board  3181 SW Sam Jackson Park Rd.,  Mail code L106-R1  Portland, OR 97239  USA | Kathryn Schuff,  M.D., MCR |
| Todd Levine, MD | Western Institutional Review Board  (WIRB)  1019 39th Avenue SE, Suite 120  Puyallup, WA 98374  USA | Glenn Veit, JD,  CIP |
| Robert Lisak, MD | Wayne State University Institutional  Review Board  87 East Canfield St., 2nd Floor  Detroit, MI 48201  USA | James Paxton,  M.D. |
| Mamatha Pasnoor, MD | University of Kansas Medical  Center Institutional Review Board  3901 Rainbow Boulevard  Kansas City, KS 66160  USA | Karen Blackwell,  MS, CIP -  Director Human  Research  Protection  Program |
| Anthony Amato, MD | Western Institutional Review Board  (WIRB)  1019 39th Avenue SE, Suite 120  Puyallup, WA 98374  USA | Glenn Veit, JD,  CIP |
| James M. Gilchrist, MD | Springfield Committee for Research  Involving Human Subjects  (SCRIHS)  201 E. Madison Street, PO Box  19664  Springfield, IL 62794  USA | Krishna Rao,  M.D., Ph.D. |
| Jonathan S. Katz, MD | Western Institutional Review Board  (WIRB)  1019 39th Avenue SE, Suite 120  Puyallup, WA 98374  USA | Glenn Veit, JD,  CIP |
| Yuebing Li, MD, PhD | Cleveland Clinic Institutional  Review Board  9500 Euclid Avenue  Cleveland, OH 44195  USA | Bridget Howard,  Esq., CIP -  Executive  Director, IRB  and Human  Research  Protections |
| Michael D. Weiss, MD | Western Institutional Review Board  (WIRB)  1019 39th Avenue SE, Suite 120  Puyallup, WA 98374  USA | Glenn Veit, JD,  CIP |
| Srikanth Muppidi, MD | Research Compliance Office,  Stanford University  3000 El Camino Real  Five Palo Alto Square, 4th Floor  Palo Alto, CA 94306  USA | Michael D.  Amylon, M.D. |
| Ericka P. Simpson, MD | Houston Methodist Research  Institute IRB  6565 Fannin St., MGJ 3  Houston, TX 77030  USA | Susan M. Miller,  M.D., MPH |
| Michael Pulley, MD | Western Institutional Review Board  (WIRB)  1019 39th Avenue SE, Suite 120  Puyallup, WA 98374  USA | Glenn Veit, JD,  CIP |
| Gil Wolfe, MD | University at Buffalo Institutional  Review Board  875 Ellicott Street, Room 5018  Buffalo, NY 14203  USA | Richard J.  Karalus, Director  of Research  Compliance |
| Dianna Quan, MD | Western Institutional Review Board  (WIRB)  1019 39th Avenue SE, Suite 120  Puyallup, WA 98374  USA | Glenn Veit, JD,  CIP |
| Richard J. Nowak, MD, MS | Western Institutional Review Board  (WIRB)  1019 39th Avenue SE, Suite 120  Puyallup, WA 98374  USA | Glenn Veit, JD,  CIP |
| Perry B. Shieh, MD, PhD | UCLA Medical Institutional Review  Board #3 (MIRB3)  10889 Wilshire Blvd, Suite 830  Los Angeles, CA 90095  USA | James McGough,  M.D. |
| Hani Kushlaf, MD, M.B.,  B.Ch. | Western Institutional Review Board  (WIRB)  1019 39th Avenue SE, Suite 120  Puyallup, WA 98374  USA | Glenn Veit, JD,  CIP |
| Gordon Smith, MD | Western Institutional Review Board  (WIRB)  1019 39th Avenue SE, Suite 120  Puyallup, WA 98374  USA | Glenn Veit, JD,  CIP |
| Gregory Sahagian, MD | Western Institutional Review Board  (WIRB)  1019 39th Avenue SE, Suite 120  Puyallup, WA 98374  USA | Glenn Veit, JD,  CIP |
| Michael Rosario-Prieto, MD | BayCare Health System IRB  4600 N Habana Ave., Suite 30  Tampa, FL 33614  USA | Jeremy  Ringewald, MD.  (Co-Chairperson)  Stephen M.  Langley, MD.  (Co-Chairperson) |
|  |  |  |

**Supplementary Table 2** Rates of TEAEs observed in AChR-Ab+ participants by concomitant use of gMG treatments

|  | Efgartigimod,  n/N (%) | Placebo, n/N (%) |
| --- | --- | --- |
| Any gMG treatment |  |  |
| At least one TEAE | 49/65 (75.4) | 53/63 (84.1) |
| At least one serious TEAE | 3/65 (4.6) | 6/63 (9.5) |
| At least one severe TEAE | 6/65 (9.2) | 7/63 (11.1) |
| At least one TEAE of special interest | 29/65 (44.6) | 21/63 (33.3) |
| At least one TEAE for which drug was withdrawn | 2/65 (3.1) | 3/63 (4.8) |
| Any steroid |  |  |
| At least one TEAE | 32/46 (69.6) | 43/51 (84.3) |
| At least one serious TEAE | 2/46 (4.3) | 4/51 (7.8) |
| At least one severe TEAE | 5/46 (10.9) | 4/51 (7.8) |
| At least one TEAE of special interest | 19/46 (41.3) | 16/51 (31.4) |
| At least one TEAE for which drug was withdrawn | 1/46 (2.2) | 3/51 (5.9) |
| Any NSIST |  |  |
| At least one TEAE | 29/40 (72.5) | 30/37 (81.1) |
| At least one serious TEAE | 2/40 (5.0) | 4/37 (10.8) |
| At least one severe TEAE | 4/40 (10.0) | 4/37 (10.8) |
| At least one TEAE of special interest | 19/40 (47.5) | 11/37 (29.7) |
| At least one TEAE for which drug was withdrawn | 1/40 (2.5) | 2/37 (5.4) |
| Any AChE inhibitor |  |  |
| At least one TEAE | 44/57 (77.2) | 49/57 (86.0) |
| At least one serious TEAE | 3/57 (5.3) | 6/57 (10.5) |
| At least one severe TEAE | 5/57 (8.8) | 7/57 (12.3) |
| At least one TEAE of special interest | 26/57 (45.6) | 17/57 (29.8) |
| At least one TEAE for which drug was withdrawn | 2/57 (3.5) | 3/57 (5.3) |

TEAEs of special interest were defined from the MedDRA system organ class “Infections and Infestations.” *AChE* acetylcholinesterase, *AChR-Ab+* acetylcholine receptor antibody–positive, *gMG* generalized myasthenia gravis, *MedDRA* Medical Dictionary for Regulatory Activities, *NSIST* nonsteroidal immunosuppressive treatment, *TEAE* treatment-emergent adverse event

**Supplementary Fig. 1**

**a**


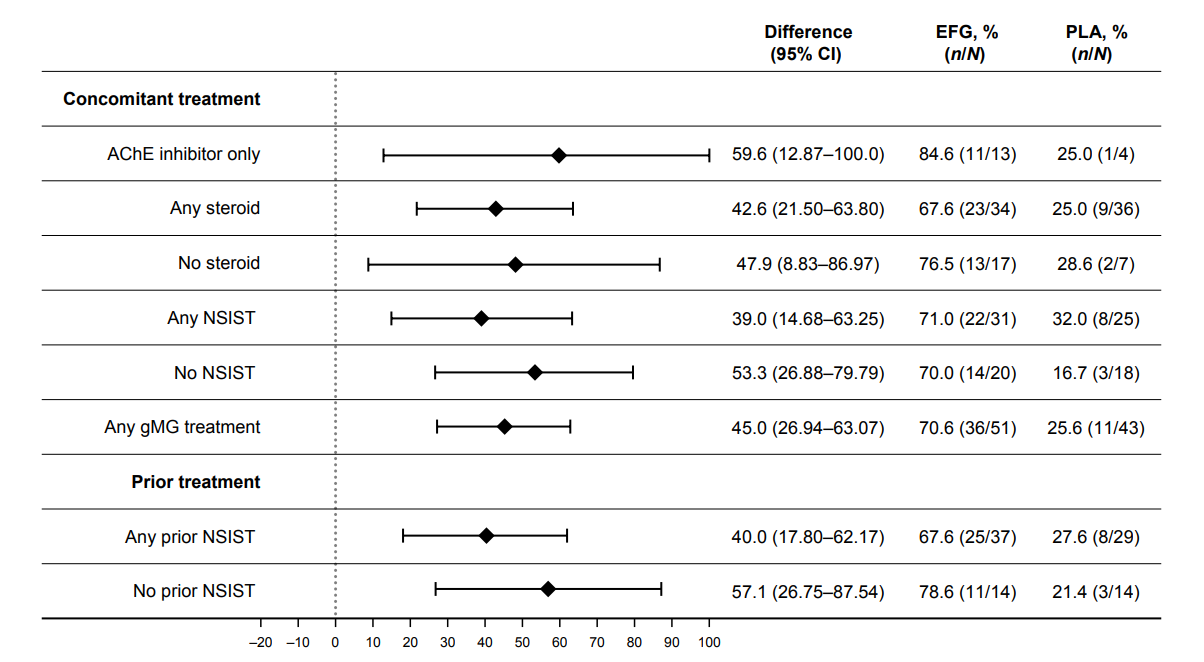


**b**

**
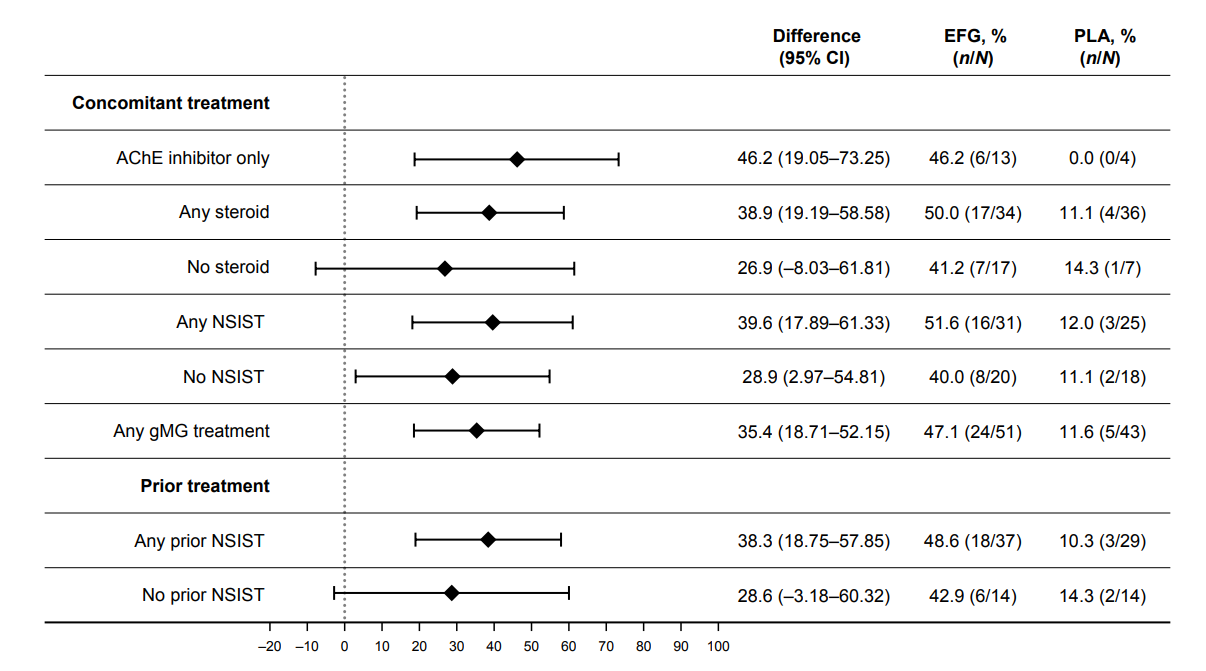
**

Proportion of **a** MG-ADL and **b** QMG responders in cycle 2 by use of gMG treatments during the original study or use of NSISTs prior to study initiation. *AChE* acetylcholinesterase, *CI* confidence interval, *EFG* efgartigimod, *gMG* generalized myasthenia gravis, *MG-ADL* Myasthenia Gravis Activities of Daily Living, *NSIST* nonsteroidal immunosuppressive treatment, *PLA* placebo, *QMG* Quantitative Myasthenia Gravis

**Supplementary** **Fig. 2**

**a**


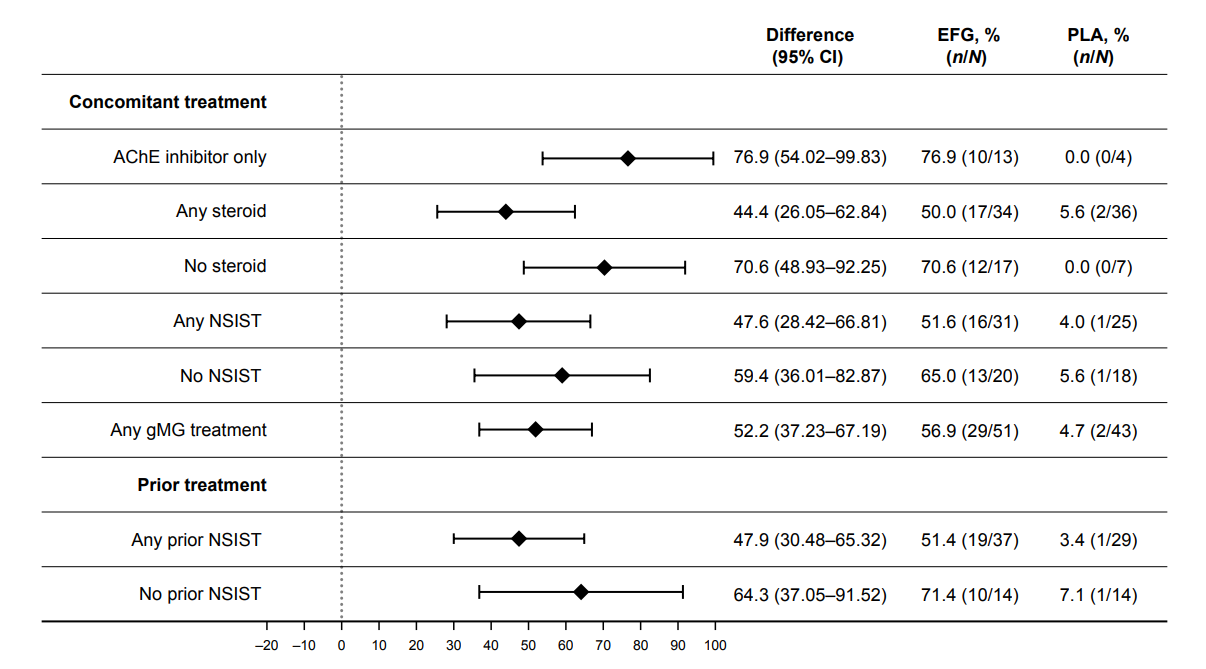


**b**


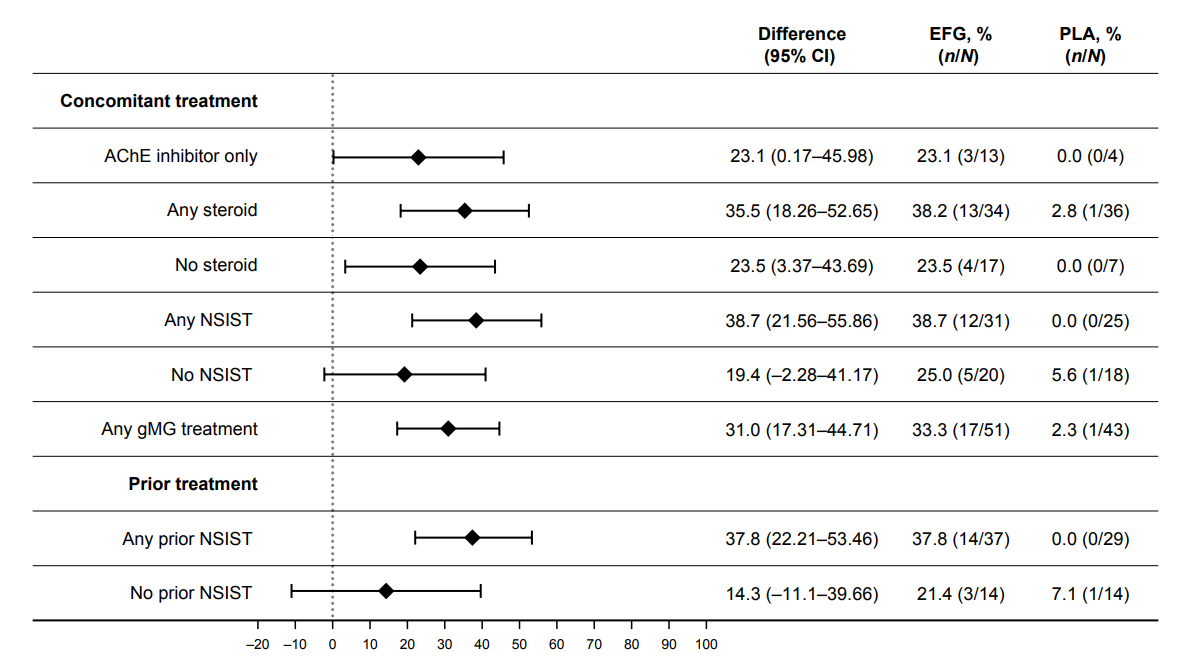


Proportion of participants who were **a** MG-ADL and **b** QMG responders in both cycle 1 and cycle 2 by use of gMG treatments during the original study or use of NSISTs prior to study initiation. *AChE* acetylcholinesterase, *CI* confidence interval, *EFG* efgartigimod, *gMG* generalized myasthenia gravis, *MG-ADL* Myasthenia Gravis Activities of Daily Living, *NSIST* nonsteroidal immunosuppressive treatment, *PLA* placebo, *QMG* Quantitative Myasthenia Gravis

**Supplementary** **Fig. 3**

**a**

**
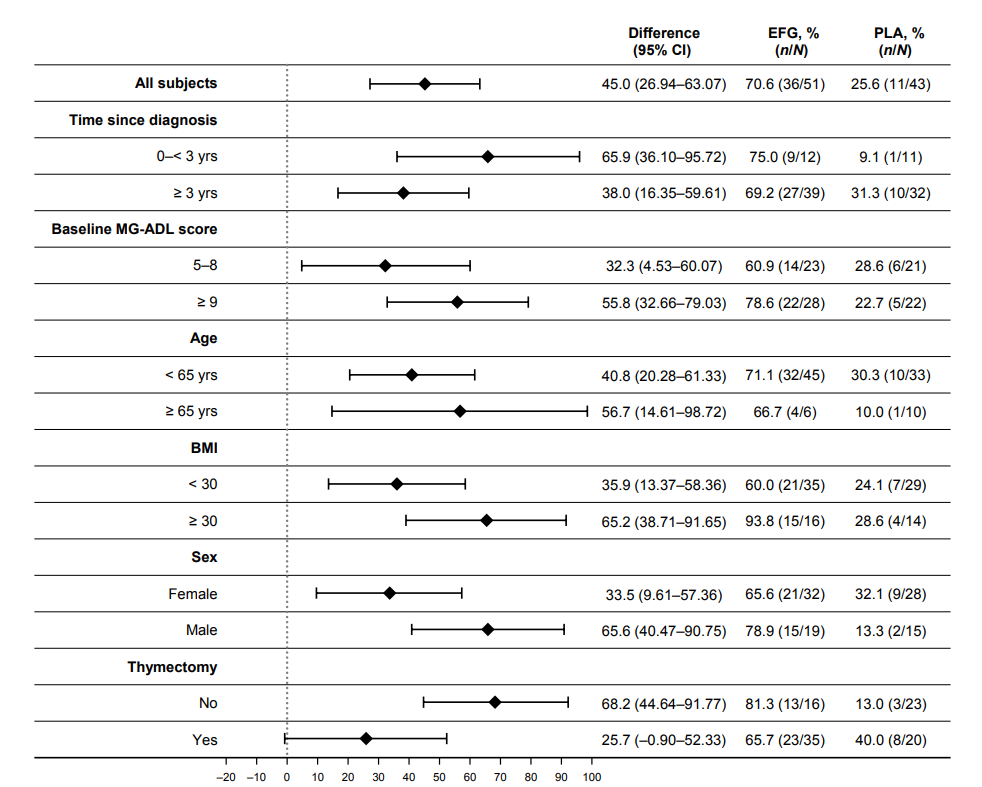
**

**b**


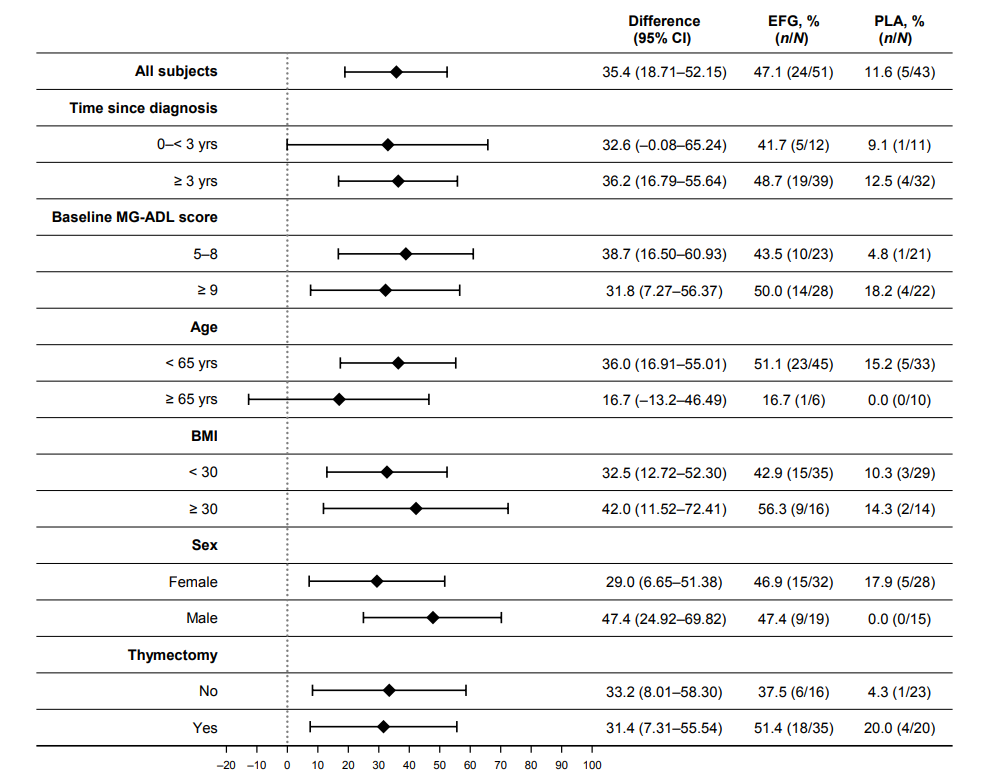


Proportion of **a** MG-ADL and **b** QMG responders in cycle 2 by baseline patient and disease characteristics. *BMI* body mass index, *CI* confidence interval, *EFG* efgartigimod, *MG-ADL* Myasthenia Gravis Activities of Daily Living, *PLA* placebo, *QMG* Quantitative Myasthenia Gravis

**Supplementary** **Fig. 4**

**a**


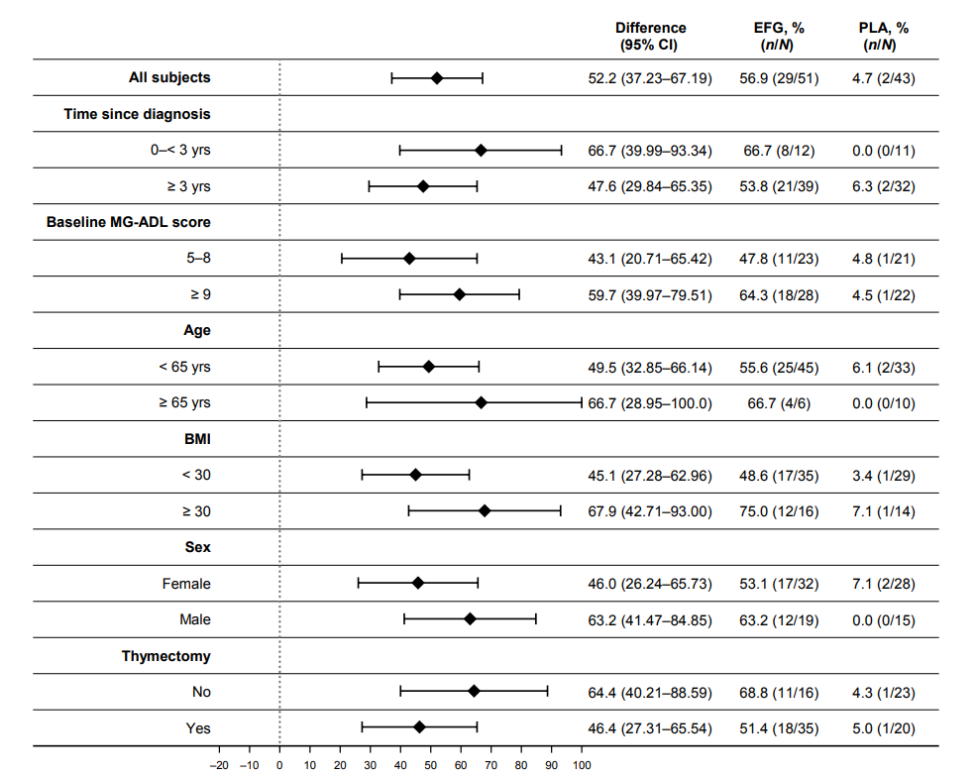


**b**

**
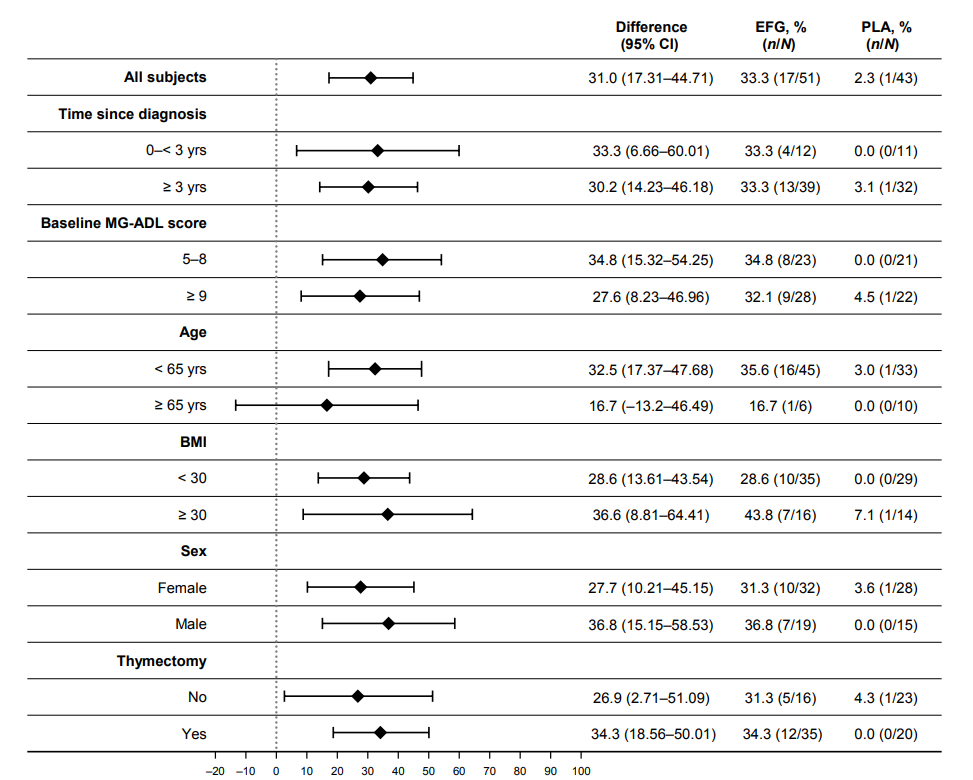
**

Proportion of participants who were **a** MG-ADL and **b** QMG responders in both cycle 1 and cycle 2 by baseline patient and disease characteristics. *BMI* body mass index, *CI* confidence interval, *EFG* efgartigimod, *MG-ADL* Myasthenia Gravis Activities of Daily Living, *PLA* placebo, *QMG* Quantitative Myasthenia Gravis
